# Modulation of u-PA, MMPs and their inhibitors by a novel nutrient mixture in human lung cancer and mesothelioma cell lines

**DOI:** 10.3892/ijo.2013.1880

**Published:** 2013-04-03

**Authors:** M. WAHEED ROOMI, TATIANA KALINOVSKY, ALEKSANDRA NIEDZWIECKI, MATTHIAS RATH

**Affiliations:** Dr Rath Research Institute, Santa Clara, CA, USA

**Keywords:** lung cancer A-549 and Calu-3, malignant mesothelioma MSTO-211H, MMP-2 and MMP-9, nutrient mixture, TIMPs, u-PA

## Abstract

Lung cancer, the most prevalent cancer worldwide and malignant mesothelioma are highly aggressive tumors that are characterized by high levels of matrix metalloproteinase (MMP)-2 and -9 secretion. Proteases play a key role in tumor cell invasion and metastasis by digesting the basement membrane and ECM components. Strong clinical and experimental evidence demonstrates association of elevated levels of u-PA and MMPs with cancer progression, metastasis and shortened patient survival. MMP activities are regulated by specific tissue inhibitors of metalloproteinases (TIMPS). Our main objective was to study the effect of a nutrient mixture (NM) on the activity of u-PA, MMPs and TIMPs on human lung and malignant mesothelioma (MM) cell lines. Human lung cancer (A-549 and Calu-3) and malignant mesothelioma (MSTO-211H) cell lines were cultured in their respective media and treated at confluence with NM at 0, 50, 100, 250, 500 and 1000 *μ*g/ml. Analysis of u-PA activity was carried out by fibrin zymography, MMPs by gelatinase zymography and TIMPs by reverse zymography. Both lung cancer cell lines expressed u-PA, which was inhibited by NM in a dose-dependent manner. However, no bands corresponding to u-PA were detected for the MSTO-211H MM cell line. On gelatinase zymography, A-549 cells showed one band corresponding to MMP-2 and induction of MMP-9 with PMA (100 ng/ml) treatment. MSTO-211H showed two bands, an intense band corresponding to MMP-2 and a faint band corresponding to MMP-9; MMP-9 was enhanced significantly with PMA treatment. NM inhibited their expression in both cell lines in a dose-dependent manner. Calu-3 showed no MMP-2 or MMP-9 expression. Activity of TIMPs was upregulated by NM in all cancer cell lines in a dose-dependent manner. Analysis revealed a positive correlation between u-PA and MMPs and a negative correlation between u-PA/MMPs and TIMPs. These findings suggest the therapeutic potential of NM in the treatment of lung and mesothelioma cancers.

## Introduction

Lung cancer (LC) is the leading cause of cancer death in men and women, accounting for 28% of all cancer deaths in 2012 (1). Approximately 226,160 new cases (14% of all new cancers) were estimated to be diagnosed in 2012 (116,470 in men and 109,690 in women). Since only 15% of lung cancer cases are diagnosed at an early stage, in the majority of lung cancer patients, metastasis has occurred by the time of diagnosis. Over half of people with lung cancer die within one year of being diagnosed. Five-year survival is only 3.5% (2). Malignant mesothelioma (MM), a rare highly aggressive tumor with no known cure, is associated with asbestos exposure. MM is characterized by aggressive local growth, invasion of vital mediastinal structures and death within 4–18 months. Metastasis has been documented in 50% of patients (3).

Metastasis occurs secondary to cancer cell detachment from the primary tumor, invasion through degraded basement membrane into the surrounding stroma and entry into and transport through the vascular or lymphatic system to distal sites such as the liver, lungs and brain and extravasation, tumor cell proliferation and angiogenesis at distal sites (4–8). Tumor cell invasion depends upon degradation of the extracellular matrix (ECM), which, when intact, acts as a barrier to block cancer cell invasion. The ECM is composed of collagen, proteoglycans, fibronectin, laminin and other glycoproteins (9–11). Two families of proteases, the matrix metalloproteinases (MMPs) and urokinase plasminogen activators (u-PA) are involved in tumor invasion and metastasis. Numerous clinical and experimental studies have demonstrated that elevated levels of u-PA and MMPs are associated with tumor growth, cancer progression, metastasis and shortened survival in patients (12–18).

MMPs, especially MMP-2 and -9 play key roles in tumor cell invasion and metastasis due to their ability to degrade type IV collagen, a major component of the ECM (11,19,20). MMP-2 and -9 are secreted in their latent zymogenic form as inactive pro-enzymes and cleaved by other MMPs or proteases into activated forms of 68, 58 and 54 kDa for MMP-2 and 84 kDa for MMP-9. Proteolytic activities of MMP-2 and -9 are inhibited by specific inhibitors, tissue inhibitors of metalloproteinases (TIMPs). Thus, a critical determinant of net proteolytic degradation is the balance between MMP and TIMP levels. Clinical studies note the association of MMP expression with progression of lung cancer (13,14) and malignant mesothelioma (15,16).

The serine protease u-PA converts plasminogen to plasmin, which is capable of promoting tumor growth and angiogenesis, degrading the ECM and basement membrane and activating pro-MMPs (21). Components of the uPA system such as u-PA, plasminogen activator inhibitor-1 (PAI-1) and urokinase-type plasminogen activator receptor (uPAR) are overexpressed in a variety of cancer types, most notably in breast cancer (22), but also in lung cancer (17,23) and malignant mesothelioma (18,24) and correlate with cancer progression, metastasis and poor prognosis. Thus the uPA system represents a potential target for anticancer strategies.

Rath and Pauling (25) proposed using nutrients such as lysine and ascorbic acid to target plasmin-mediated connective tissue degradation as a universal approach to tumor growth and expansion. Binding to plasminogen active sites, lysine blocks plasminogen activation into plasmin by tissue plasminogen activator (t-PA). Thus it modulates the plasmin-induced MMP activation cascade (26). Subsequent studies confirmed this approach and resulted in identifying a novel formulation composed of lysine, ascorbic acid, proline and green tea extract and other micronutrients (NM), which has shown significant anticancer activity against a large number (∼40) of cancer cell lines, blocking cancer growth, tissue invasion and MMP expression both *in vitro* and *in vivo*(27). In this study, we focused on the modulating effect of NM on the activities of MMP-2 and -9, TIMPs and u-PA in human lung cancer (A-549 and Calu-3) and malignant mesothelioma (MSTO-211H) cell lines.

## Materials and methods

### Materials

Human lung cancer cells A-549 and Calu-3 and malignant mesothelioma MSTO-211H, along with their culture media Ham F-12 and RPMI were obtained from ATCC. Antibiotics, penicillin and fetal bovine serum (FBS), were obtained from Gibco (BRL, Long Island, NY). Twenty-four well tissue culture plates were obtained from Costar (Cambrdige, MA). Gelatinase zymography was performed in 10% Novex pre-cast SDS polyacrylamide gel (Invitrogen Inc.) with 0.1% gelatin in non-reducing conditions. The nutrient mixture (NM), prepared by VitaTech (Hayward, CA) was composed of the following ingredients in the relative amounts indicated: vitamin C (as ascorbic acid and as Mg, Ca and palmitate ascorbate) 700 mg; L-lysine 1000 mg; L-proline 750 mg; L-arginine 500 mg; N-acetyl cysteine 200 mg; standardized green tea extract (80% polyphenol) 1000 mg; selenium 30 *μ*g; copper 2 mg; manganese 1 mg. All other reagents used were of high quality and were obtained from Sigma, unless otherwise indicated.

### Cell cultures

The LC (A-549 and Calu-3) and MM (MSTO-211H) cell lines were grown in Ham F-12 and RPMI media, supplemented with 10% FBS, penicillin (100 U/ml) and streptomycin (100 *μ*g/ml) in 24-well tissue culture plates. The cells were plated at a density of 1×10^5^ cells/ml and grown to confluency in a humidified atmosphere at 5% CO_2_ at 37°C. Serum-supplemented media were removed and the cell monolayer was washed once with PBS with the recommended serum-free media. The cells were treated with the nutrient mixture, dissolved in media and tested at 0, 50, 100, 250, 500 and 1000 *μ*g/ml in triplicate at each dose. Parallel sets of cultures were treated with PMA (100 ng/ml) for induction of MMP-9. Control and PMA treatments were done in triplicates. The plates were then returned to the incubator. The conditioned media were collected separately, pooled and centrifuged at 4°C for 10 min at 3,000 rpm to remove cells and cell debris. The supernatant was collected and used to assess for u-PA activity (by fibrin zymography on 10% SDS-PAGE gels containing fibrinogen and plasminogen), MMP-2 and -9 (by gelatinase zymography) and TIMPs (by reverse zymography).

### Fibrin zymography

Fibrin zymography was used to analyze u-PA activity on 10% SDS-PAGE gels containing fibrinogen (5.5 mg/ml) and plasminogen (50 *μ*g/ml). After electrophoresis, the gels were washed twice with 2.5% Triton X-100 for 30 min. The gels were then incubated overnight at 37°C with 0.1% glycine buffer pH 7.5 and then stained with 0.5% Coomassie Brilliant Blue R250 and destained. Electrophoresis of u-PA and t-PA were conducted for comparison. Fibrin zymograms were scanned using CanoScan 9950F Canon Scanner.

### Gelatinase zymography

Gelatinase zymography was performed in 10% NOVEX Pre-Cast SDS polyacrylamide gel (Invitrogen Corp.) in the presence of 0.1% gelatin under non-reducing conditions. Culture media (20 *μ*l) were mixed with sample buffer and loaded for SDS-PAGE with Tris-glycine SDS buffer as suggested by the manufacturer (Novex). Samples were not boiled before electrophoresis. Following electrophoresis the gels were washed twice in 2.5% Triton X-100 for 30 min at room temperature to remove SDS. The gels were then incubated at 37°C overnight in substrate buffer containing 50 mM Tris-HCl and 10 mM CaCl_2_ at pH 8.0 and stained with 0.5% Coomassie Blue R250 in 50% methanol and 10% glacial acetic acid for 30 min and destained. Upon renaturation of the enzyme, the gelatinases digest the gelatin in the gel and give clear bands against an intensely stained background. Protein standards were run concurrently and approximate molecular weights were determined by plotting the relative mobilities of known proteins.

### Reverse zymography

TIMPs were analyzed by reverse zymography on 15% SDS gels containing serum-free conditioned medium from cells. After electrophoresis the gels were washed twice with 2.5% Triton-X for 30 min at room temperature to remove SDS. The gels were then incubated at 37°C overnight in 50 mM Tris-HCl and 10 mM Ca Cl_2_ at pH 7.6 and stained with 0.5% Coomassie Blue R25, destained and scanned.

### Scanning of gelatinase and fibrin zymograms

Gelatinase, reverse and fibrin zymograms were scanned using CanoScan 9950F Canon scanner at 300 dpi. The intensity of the bands was evaluated using the pixel-based densitometer program Un-Scan-It, Version 5.1, 32-bit, by Silk Scientific Corp. (Orem, UT, USA), at a resolution of 1 Scanner Unit (1/100 of an inch for an image that was scanned at 100 dpi). The pixel densitometer calculates the optical density of each pixel (values 0–255) using the darkly stained background of the gel as a pixel value of 0. A logarithmic optical density scale was used since the optical density of films and gels is logarithmically proportional to the concentration. The pixel densitometer sums the optical density of each pixel to give a band’s density. In all graphs, band densities were reported as percentages of the sums of all pixels in a given lane (treatment) of a gel.

### Statistical analysis

Pearson’s correlation coefficient was determined between mean MMP-2, u-PA and TIMP-2 expressions of lung cancer cell line A-549, mean u-PA and TIMP-2 expressions of lung cancer Calu-3 and between mean MMP-9 and TIMP-2 expression of malignant mesothelioma cell line MSTO-211H using MedCalc Software (Mariakerke, Belgium).

## Results

### Effect of NM on u-PA activity in human lung cancer and mesothelioma cell lines

**Figure 1:**
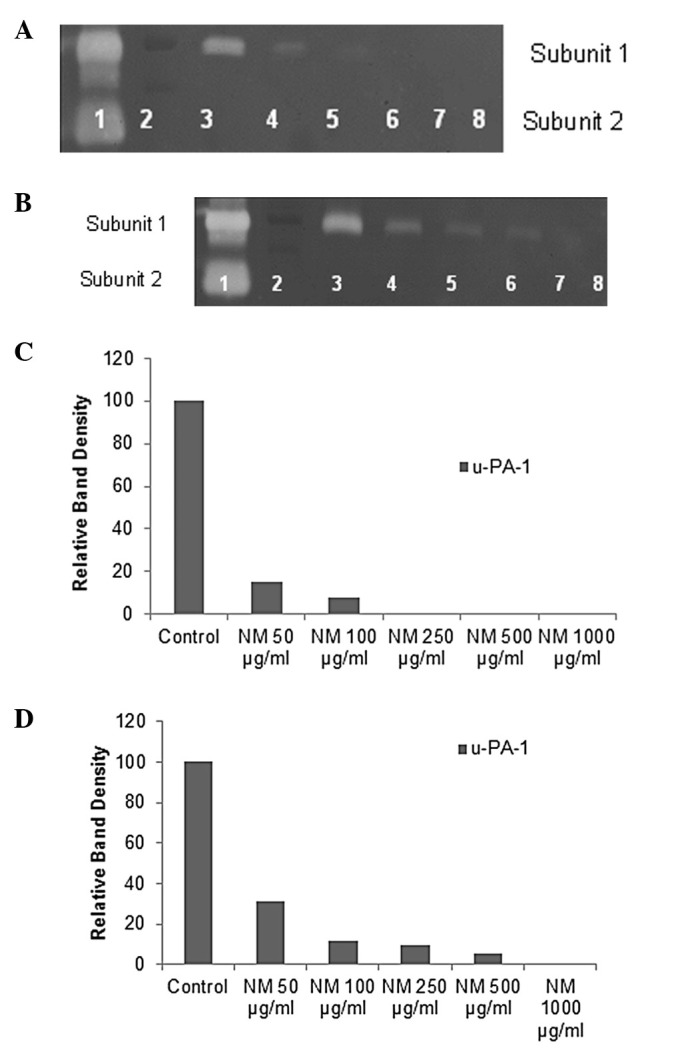
Effect of NM on lung cancer cell lines A-549 and Calu-3 u-PA expression. Fibrin zymograms of A-549 (A), Calu-3 (B) u-PA expression. Lane 1, u-PA; lane 2, markers; lane 3, control; lanes 4–8, NM 50, 100, 250, 500 and 1,000 *μ*g/ml. Densitometric analyses of A-549 (C) and Calu-3 (D) u-PA expression.

Activity of u-PA was detected in both lung cancer cell lines showing one band corresponding to subunit 1 (55 kDa). However, no bands corresponding to u-PA were detected for mesothelioma cell line. NM exerted dose response inhibition with virtual block of u-PA activity at 250 *μ*g/ml in A-549 cells (linear trend R^2^=0.563) and 1000 *μ*g/ml in Calu-3 cells (linear trend R^2^=0.680) (see Fig. 1 for respective fibrin zymograms and densitometry analyses).

### Effect of NM on MMP-2 and -9 expression by human lung cancer and mesothelioma cell lines

**Figure 2:**
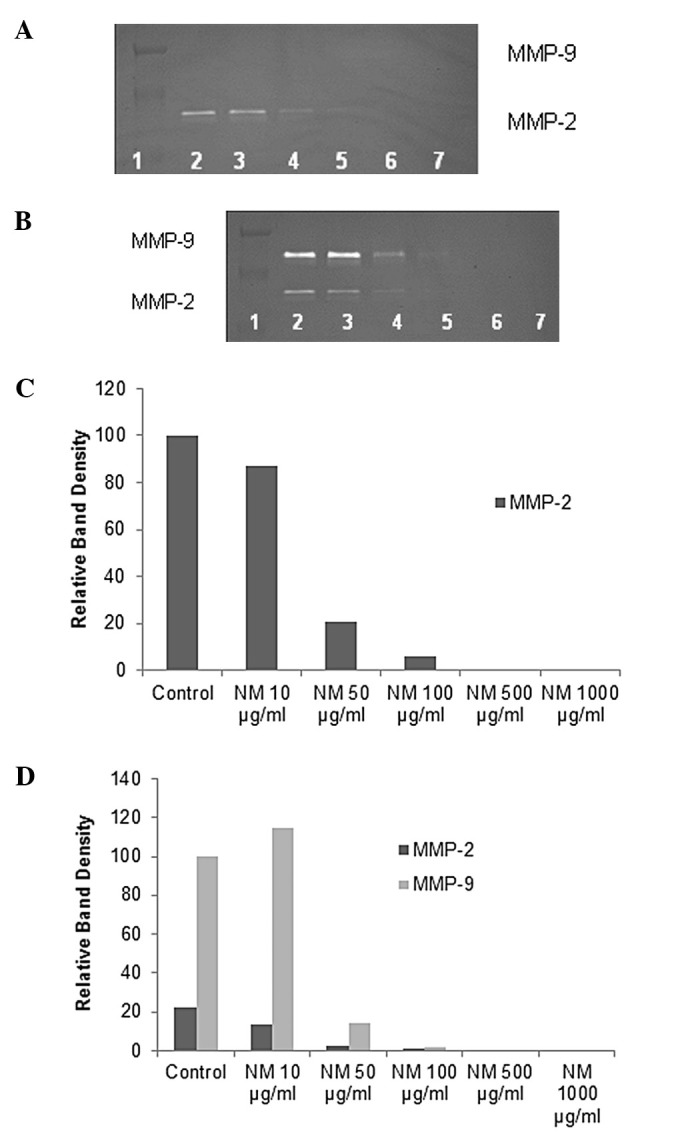
Effects of NM on lung cancer cell lines A-549 MMP-2 and -9 expression. Gelatinase zymograms of normal A-549 cell MMP-2 secretion (A) and PMA-treated A-549 cell MMP-2 and -9 secretion (B). Lane 1, markers; lane 2, control; lanes 3–7, NM 50, 100, 250, 500 and 1,000 *μ*g/ml. Densitometric analyses of normal A-549 cell MMP-2 secretion (C) and PMA-treated A-549 MMP-2 and -9 secretion (D).

**Figure 3:**
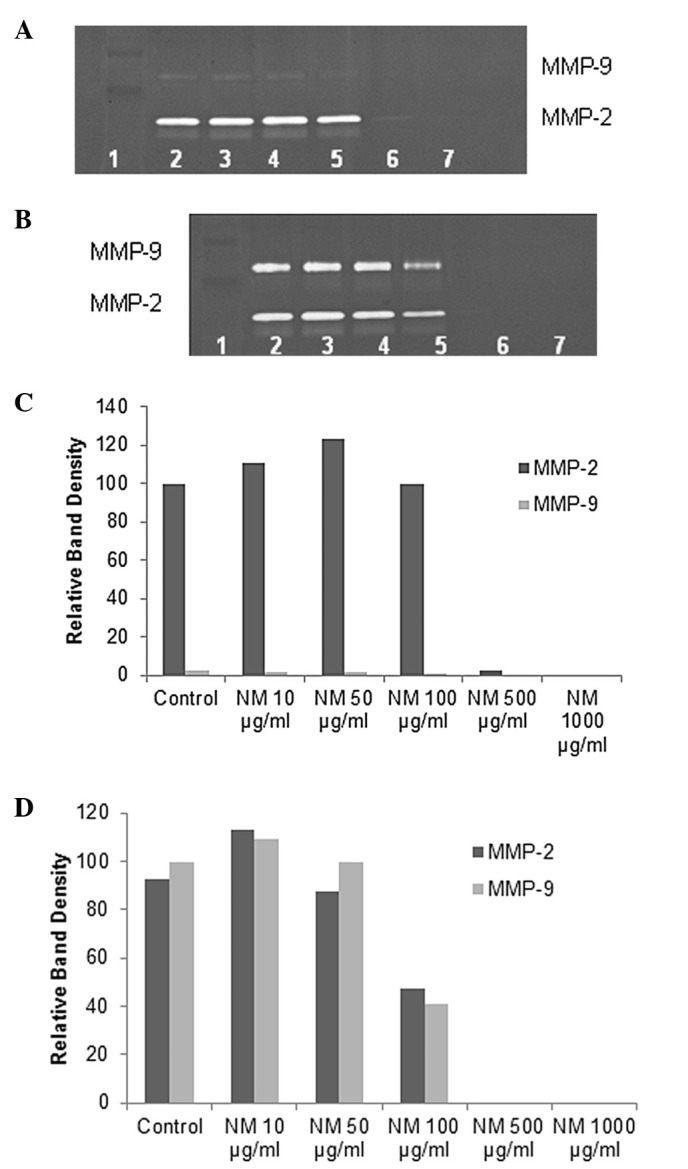
Effects of NM on malignant mesothelioma MSTO-211H MMP-2 and -9 expression. Gelatinase zymograms of normal MSTO-211H (A) and PMA-treated MSTO-211H (B) cell MMP-2 and -9 secretion. Lane 1, markers; lane 2, control; lanes 3-7, NM 50, 100, 250, 500 and 1,000 *μ*g/ml. Densitometric analysis of normal (C) and PMA-treated (D) MSTO-211H MMP-2 and -9 secretion.

On gelatinase zymography, a band corresponding to MMP-2 was detected in normal A-549 cells and strong induction of MMP-9 with PMA (100 ng/ml) treatment. NM inhibited MMP-2 and -9 in a dose-dependent manner with total block at 500 *μ*g/ml (linear trend R^2^=0.781 for MMP-2 and 0.722 for MMP-9). Mesothelioma MSTO-211H cells showed two bands, an intense band corresponding to MMP-2 and a faint band corresponding to MMP-9, which was significantly enhanced with PMA treatment. NM inhibited MMP expression, with complete block of MMP-2 and -9 at 500 *μ*g/ml (linear trend R^2^ = 0.849 for MMP-2 and R^2^=0.853 for MMP-9) (see Figs. 2 and 3 for gelatinase zymograms and densitometry analyses).

### Effect of NM on TIMP activity in human lung and cancer and mesothelioma cell lines

**Figure 4:**
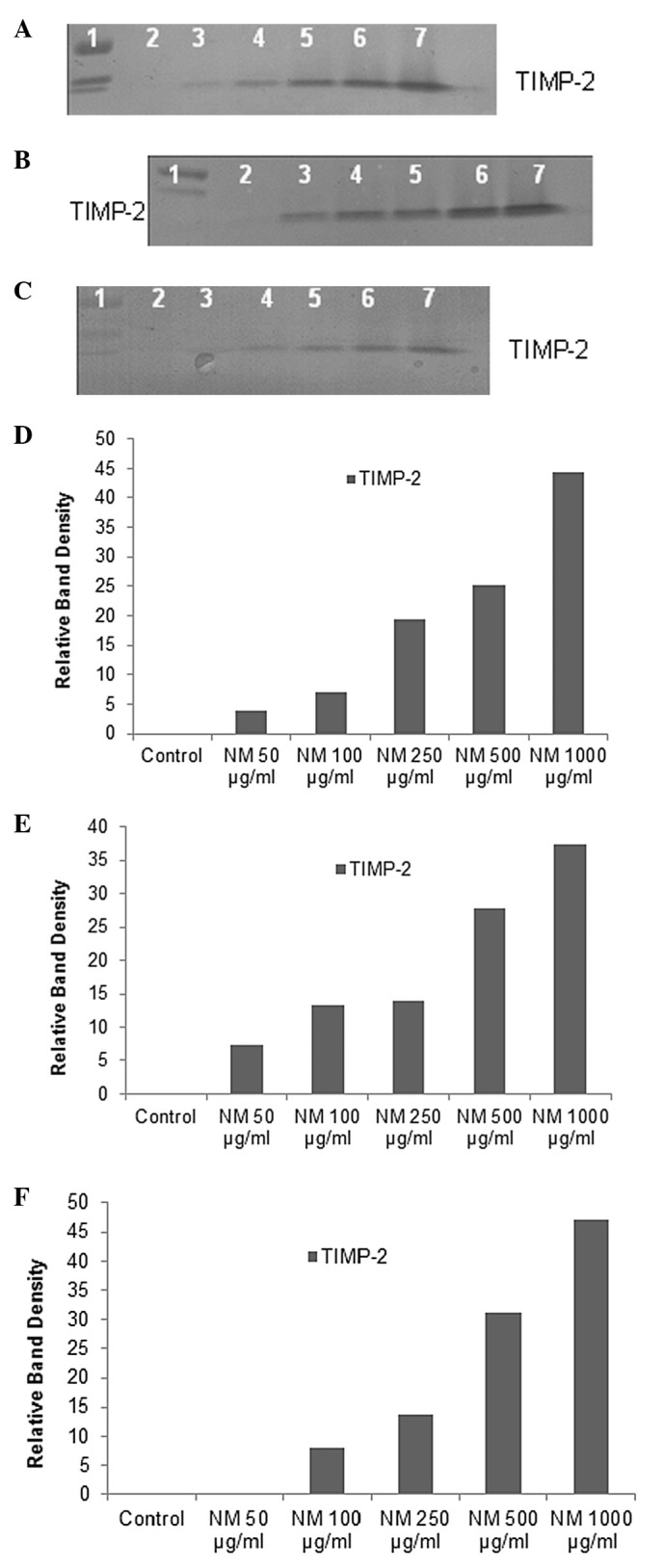
Effect of NM on lung cancer cell lines A-549 and Calu-3 and malignant mesothelioma MSTO-211H TIMP-2 expression. Reverse zymograms of A-549 (A) Calu-3 (B) and MSTO-211H (C) TIMP-2 expression. Lane 1, markers; lane 2, control; lanes 3–7, NM 50, 100, 250, 500 and 1,000 *μ*g/ml. Densitometric analyses of A-549 (D), Calu-3 (E) and MSTO-211H (F) TIMP-2 expression.

Reverse zymography revealed upregulation of TIMP-2 activity with NM treatment in all cancer cell lines in a dose-dependent manner. Minimum activity was expressed at 50 and maximum at 1,000 *μ*g/ml NM (see Fig. 4 for respective reverse zymograms and densitometry analyses).

### Correlation between lung cancer and mesothelioma u-PA, TIMP-2 and MMP expressions

**Figure 5:**
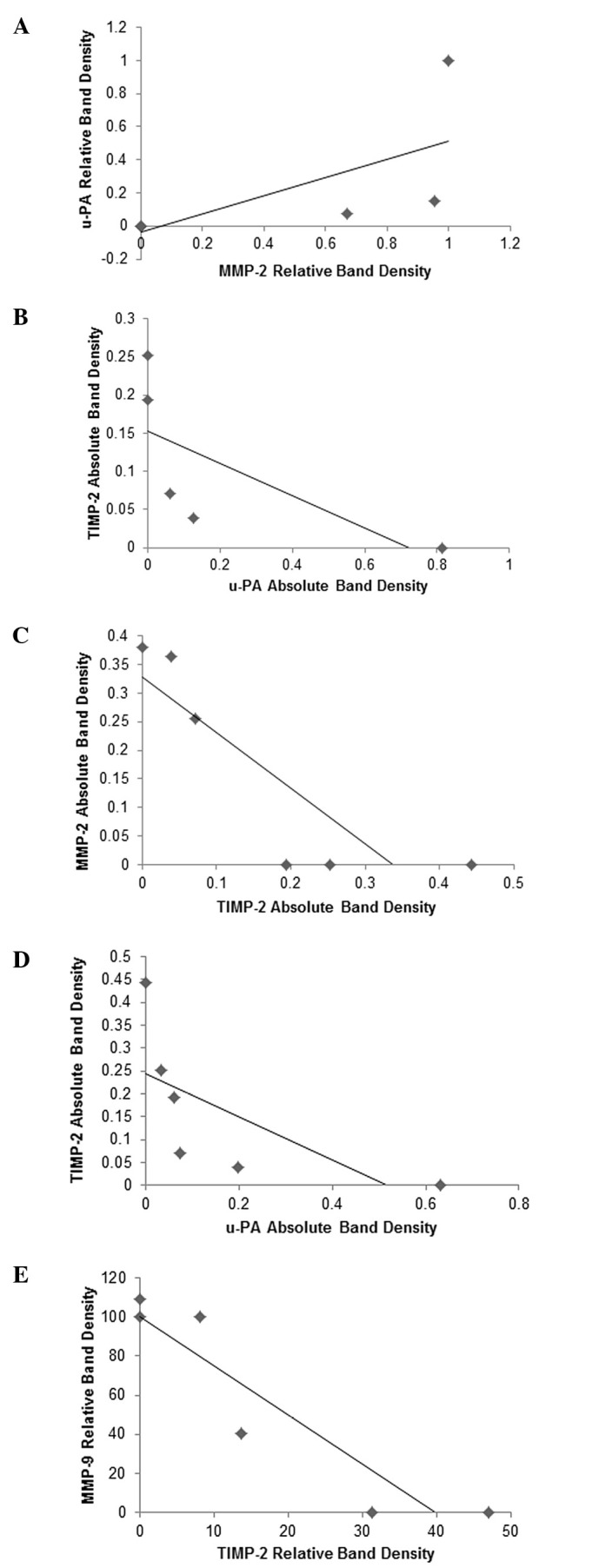
(A) Correlation between the effects of NM on lung cancer A-549 u-PA and TIMP-2 expression. (B) Correlation between the effects of NM on lung cancer A-549 TIMP-2 and u-PA expression. (C) Correlation between the effects of NM on lung cancer A-549 MMP-2 and TIMP-2 expression. (D) Correlation between the effects of NM on lung cancer Calu-3, u-PA and TIMP-2 expression. (E) Correlation between the effects of NM on MM MSTO-211H MMP-9 and TIMP-2 expression.

Analysis revealed a positive correlation between NM-treated lung cancer cell line A-549 u-PA and MMP-2 expressions, as shown in Fig. 5A, with a correlation coefficient r= 0.679. Negative correlations were found between the expressions of A-549 u-PA and TIMP-2 (correlation coefficient r=−0.685, Fig. 5B) and MMP-2 and TIMP-2 (correlation coefficient r=−0.862, Fig. 5C). Negative correlations were found between Calu-3 expression of TIMP-2 and u-PA (correlation coefficient r=−0.674, Fig. 5D) and between MSTO-211H expression of TIMP-2 and MMP-9 (correlation coefficient r=−0.925, Fig. 5E).

## Discussion

Tumor cell invasion requires the critical steps of cell attachment, degradation of the ECM and migration through the disrupted matrix. The two families of proteases, matrix metalloproteinases and urokinase plasminogen activators play key roles in tumor cell invasion. Experimental studies have demonstrated the role of urokinase plasminogen, especially cell surface u-PA, as an initiator of ECM proteolysis and associated tumor cell invasion (26). The protease u-PA converts plasminogen to plasmin, which is capable of promoting tumor growth and angiogenesis, degrading the ECM and basement membrane and activating pro-MMPs (21). Duffy *et al* first reported the prognostic value of u-PA in breast cancer patients, showing a positive correlation between high levels of u-PA and cancer progression (22). Overexpression of u-PA in lung cancer has been correlated with cancer progression, metastasis and poor prognosis (17). Though the pathogenesis of the aggressive neoplasm malignant mesothelioma remains unclear, the invasiveness of MM cells has been correlated with disordered fibrin turnover and pro-coagulant and fibrinolytic activity (17). MM is locally invasive and tumor cells are often surrounded by a dense collagenous stroma; neoplastic spread is thought to be facilitated by fibrin and plasminogen activators, which control inflammatory cell traffic through the tumor matrix, as well as by promoting neovascularization (24). Idell *et al* found that fibrinolytic activity of human pleural mesothelioma cell line MS-1 was mainly due to urokinase and was responsive to cytokine stimulation (24). Matrix metalloproteinases, especially MMP-2 and -9 play pivotal roles in tumor cell invasion and metastasis due to their ability to degrade type IV collagen, a major component of the ECM. Overproduction of MMPs, especially MMP-2 and -9 has been associated with a more aggressive behavior of lung cancer and mesothelioma (13–16).

Our study demonstrated that the specific mixture of nutrients tested significantly inhibited u-PA secretion in lung cancer cell A-549 and Calu-3 (malignant mesothelioma cell line MSTO-211H was not found to secrete u-PA in this study). Furthermore, the NM demonstrated dose-dependent decrease in MMP secretion and increase in TIMP-2 secretion by both lung cancer A-549 and mesothelioma cell lines. As expected, a significant positive correlation was found between the secretion of u-PA and MMP-2 and a significant negative correlation between u-PA and TIMP-2 secretion by NM treatment of lung cancer A-549 cells. As anticipated, a significant negative correlation was found between MMP-9 and TIMP-2 secretion by MSTO-211H cell line. Furthermore, a previous study demonstrated significant correlation between NM inhibition of Matrigel invasion and NM modulation of the MMP-2 and -9 activity of the lung cancer and MM cell lines studied (28). A significant negative correlation was found between inhibition of NM modulation of Matrigel invasion and MMP-2 secretion with lung cancer A-549 (r=−0.905). For malignant mesothelioma MSTO-211H cells, a significant negative correlation (r=−0.955 was found between inhibition of NM modulation of Matrigel invasion and MMP-9 secretion. Previous *in vivo* studies of the effects of NM on lung cancer growth and metastasis support these results in that it demonstrated significant (47%, p<0.0001) inhibition of A-549 xenograft tumor growth in nude mice (29).

In contrast to the associated toxicity and limited efficacy of standard cancer chemotherapy and radiation therapy, the efficacy and safety of dietary and botanical natural compounds in cancer prevention has been extensively documented (30). The nutrient mixture was formulated by selecting nutrients that act on critical physiological targets in cancer progression and metastasis, as documented in both clinical and experimental studies. Combining these micronutrients expands metabolic targets, maximizing biological impact with lower doses of components. A previous study of the comparative effects of NM, green tea extract and EGCG on inhibition of MMP-2 and -9 secretion of different cancer cell lines with varying MMP secretion patterns, revealed the superior potency of NM over GTE and EGCG at equivalent doses (31). These results can be understood from the more comprehensive treatment offered by the combination of nutrients in NM over individual components of NM since MMP-2 and -9 are mediated by differential pathways.

Optimal ECM structure depends upon adequate supplies of ascorbic acid and the amino acids lysine and proline to ensure proper synthesis and hydroxylation of collagen fibers. In addition, lysine contributes to ECM stability as a natural inhibitor of plasmin-induced proteolysis (25,32). Manganese and copper are also essential for collagen formation. There is considerable documentation of the potency of green tea extract in modulating cancer cell growth, metastasis, angiogenesis and other aspects of cancer progression (33–39). N-acetyl cysteine and selenium have demonstrated inhibition of tumor cell MMP-9 and invasive activities, as well as migration of endothelial cells through ECM (40–42). Ascorbic acid demonstrates cytotoxic and antimetastatic actions on malignant cell lines (43–47) and cancer patients have been found to have low levels of ascorbic acid (48,49). Low levels of arginine, a precursor of nitric oxide (NO), can limit the production of NO, which has been shown to predominantly act as an inducer of apoptosis (50).

In conclusion, the NM demonstrated potent anticancer activity by targeting primary mechanisms responsible for the aggressive spread of lung cancer and malignant mesothelioma. In this *in vitro* study, the NM significantly inhibited secretion of u-PA and increased secretion of TIMP-2 in lung cancer cell lines A-549 and Calu-3, as well as decreased MMP-2 secretion in A-549 cells, suggesting its potential in modulating lung cancer invasion and metastasis. Malignant mesothelioma MSTO-211H cells did not secrete u-PA; however, MMP-9 secretion by MSTO-211H was inhibited by NM and secretion of TIMP-2 was enhanced by NM. NM inhibition of MMP secretion was found to be correlated significantly with Matrigel invasion of lung cancer A-549 and MSTO-211H cells. Furthermore, use of the nutrient mixture would not pose any toxic effect clinically, especially in the relevant doses, as *in vivo* safety studies demonstrate. An *in vivo* toxicology study showed that NM had no adverse effects on vital organs (heart, liver and kidney), or on the associated functional serum enzymes (51).

## Figures and Tables

**Table I:** Overview of MMP-2 and -9, u-PA and TIMP-2 expression of lung cancer and mesothelioma cell lines.

Cancer cell line	MMP-2	MMP-9	u-PA	TIMP-2
Lung A-549	+	With PMA tx	+	+
Lung Calu-3	−	−	+	+
MM MSTO-211H	+	+	−	+
